# *Leishmania infantum* eukaryotic initiation factor 4A modulates *in vitro* functional activity of human neutrophils

**DOI:** 10.3389/fimmu.2026.1873341

**Published:** 2026-09-01

**Authors:** Rafeh Oualha, Yosser Zina Abdelkrim, Mourad Barhoumi, Sonia Ben Hamouda, Imen Bassoumi-Jamoussi, Makram Essafi, Melika Ben Ahmed, Khadija Essafi-Benkhadir, Ikram Guizani

**Affiliations:** 1 Laboratory of Molecular Epidemiology and Experimental Pathology, Institut Pasteur de Tunis, Université de Tunis El Manar, Tunis, Tunisia; 2 Laboratory of Transmission, Control and Immunobiology of Infections, Institut Pasteur de Tunis, Université de Tunis El Manar, Tunis, Tunisia

**Keywords:** cellular effectors, excreted/secreted *Leishmania* products, innate immunity, LieIF (Leishmania infantum eukaryotic initiation factor 4A), neutrophils

## Abstract

Leishmaniases are a group of neglected tropical diseases caused by *Leishmania* parasites which virulence mainly relies on its modulation of the host immune response. Neutrophils, the first recruited innate immune cells at the site of infection, are key determinants of the early parasite control and disease outcome. However, their functional activation in response to parasite-derived molecules remains poorly understood. In this study, we investigated the effect of the *Leishmania infantum* Eukaryotic Initiation Factor 4A (LieIF), an excreted/secreted protein, on human neutrophils. Stimulation with recombinant LieIF protein induced a strong pro-inflammatory neutrophil response characterized by increased ROS production, degranulation, late apoptosis and enhanced secretion of IL-8, TNF-α, IL-1β and IL-6 cytokines associated with a reduced production of the anti-inflammatory cytokine TGF-β1. In contrast, IL-10, IFN-γ and IL-12p70 were not detected. Furthermore, LieIF induced the activation of p38 MAPK but had no effect on AKT, ERK1/2 and JNK kinases. Finally, inhibition of p38, using a specific pharmacological inhibitor, resulted in lesser production of TNF-α, IL-1β, IL-6 and IL-8 cytokines, confirming the functional contribution of the p38 MAPK in LieIF-induced neutrophil activation. These findings identify LieIF as a potent parasite-derived inflammatory protein that drives neutrophil activation via p38 kinase, highlighting its role in early innate immune response and its potential relevance for the development of novel therapeutic and vaccine strategies.

## 1 Introduction

Leishmaniases are a group of neglected tropical diseases caused by *Leishmania* parasites, transmitted by sandfly vectors. These diseases affect millions of individuals worldwide, particularly in endemic regions, and exhibit highly variable clinical outcomes ranging from self-limiting cutaneous lesions to life-threatening visceral infections. The severity and progression of disease are largely determined by the host immune response, which is shaped by both the parasite’s virulence factors and the characteristics of innate and adaptive immune cells. Early interactions between the parasite and innate immune cells, particularly neutrophils, play a crucial role in defining the infection outcome (1–3).

Neutrophils are among the first immune cells recruited to the site of *Leishmania* inoculation and serve as key mediators of early parasite control. They can rapidly phagocytose promastigotes, generate reactive oxygen species (ROS), release neutrophil extracellular traps (NETs), and secrete chemokines to orchestrate immune recruitment (4). Parasites have therefore developed several strategies to manipulate neutrophil functions such as delaying apoptosis. The specific impact of parasite-derived molecules on neutrophil modulation remains largely unexplored. Among parasite-derived factors reported to interfere with neutrophil functions, the major surface glycoconjugate lipophosphoglycan (LPG) has been shown to induce neutrophil extracellular trap (NET) formation and to modulate oxidative responses (5). In addition, *Leishmania* excretory/secretory products (ESPs), including enzymes such as 3′-nucleotidase/nuclease and chemotactic factors, have been implicated in modulating the local immune microenvironment and influencing early host–parasite interactions (6).

Furthermore, *Leishmania*-derived exosomes, a class of extracellular vesicles containing proteins, lipids, and nucleic acids (DNA, mRNA and microRNA), have been shown to play an important role in host–parasite communication and immune modulation (7–11). Collectively, these parasite-derived components highlight the complexity of host–parasite cross talk during the early infection stage. Among them, the *Leishmania* eukaryotic initiation factor 4A (LeIF) has itself been identified as a component of the *Leishmania* secretome and exosome fractions, suggesting that it may be delivered to host immune cells through vesicular as well as soluble secretion pathways (12–14). Indeed, LeIF, a 45.3 kDa cytoplasmic protein of 403 amino acids and belonging to the highly conserved DEAD-box family of RNA helicases (15, 16), has attracted considerable attention due to its marked immunomodulatory properties. This protein is highly conserved across *Leishmania* species and is constitutively expressed in both promastigote and amastigote developmental stages (17, 18). However, its physiological concentration that could be released during natural infection is currently unknown. LeIF was initially identified through the screening of a genomic DNA library from *L. braziliensis* using sera from patients with mucocutaneous leishmaniasis (17). Sequence analysis has demonstrated that LeIF shares significant evolutionary conservation, with approximately 54.6% identity to yeast eIF4A and 56.1% to its human and murine homologs (15). Previous studies, including those from our team have demonstrated that recombinant LeIF induces strong pro-inflammatory and Th1-oriented immune responses in monocytes, macrophages, dendritic cells, and peripheral blood mononuclear cells (17–24). These properties have highlighted its potential relevance as a novel candidate for immunotherapeutic and vaccine strategies against leishmaniases. Despite the extensive investigations described above, the impact of LeIF on neutrophil activation remains largely unexplored. Since neutrophils are the first responders to *Leishmania* infection, understanding how this conserved parasite-derived molecule influences neutrophil activation is essential to better define the early immune events that shape disease outcome.

In this study, we hypothesized that LieIF acts as a potent secreted immunomodulator capable of directly modulating neutrophil effector functions. To address this, we evaluated the effects of recombinant LieIF protein on primary human neutrophils through assessing key responses, including ROS production, degranulation, cell survival and cytokine production. Furthermore, we investigated the underlying molecular mechanisms, focusing on the mitogen-activated protein kinase (MAPK) and AKT signaling pathways in LieIF-treated neutrophils.

## 2 Materials and methods

### 2.1 Reagents and materials

Recombinant *L. infantum* LeIF protein (rLieIF) was produced in *Escherichia (E). coli* BL21(DE3) and purified as described in section 2.3. All culture media and biochemical reagents were purchased from Sigma-Aldrich unless otherwise specified.

### 2.2 Ethics statement

Human peripheral blood samples were obtained from healthy volunteer adult donors after written informed consent. Sampling and experimental procedures were approved by the Institutional Ethics Committee of the Institut Pasteur de Tunis (2018/07/I/LR11IPT04).

### 2.3 Expression and purification of recombinant LieIF protein

The LieIF coding sequence was previously cloned into the pET-22b(+) expression vector (15). The recombinant plasmid was used to transform chemically competent *E. coli* BL21(DE3) cells. Transformed cells were grown in Luria Broth (LB) medium supplemented with 50 µg/mL ampicillin at 37 °C until reaching an OD_600_ of 0.4. Protein expression was induced by adding isopropyl β-D-1-thiogalactopyranoside (IPTG) to a final concentration of 0.4 mM for 3 h at 30 °C. Cells were harvested and resuspended in lysis buffer (20 mM Tris-HCl pH 8.0, 300 mM NaCl, 10 mM imidazole) containing 2 mM PMSF (PhenylMethylSulfonyl Fluoride) and Lysozyme (10 mg/mL). The lysate was incubated on ice for 30 min. Cell disruption was completed by sonication (4 × 20 s pulses), followed by centrifugation at 15,000 rpm for 30 min at 4 °C to collect the soluble fraction.

The recombinant His-tagged LieIF protein was purified using nickel-affinity chromatography. Briefly, the supernatant was loaded onto the nickel-charged chelating sepharose column previously washed with 40 mM imidazole before being re-equilibrated with 10 mM Tris-HCl. The column was then treated with 10 mM Tris-HCl containing 0.5% of the detergent ASB (amidosulfobetaine-14) to remove endotoxins and was washed with 10 volumes of 10 mM Tris-HCl to eliminate excess detergent. Recombinant His-tagged LieIF was eluted using lysis buffer supplemented with 100 mM imidazole. All purification steps were performed at 4 °C. Fractions containing the recombinant protein were pooled and desalted to remove excess imidazole using a PD-10 desalting column (Amersham, GE Healthcare, Velizy-Villacoublay, France) and PBS (1x) containing protease inhibitors. The purified protein was aliquoted and stored in 50% glycerol at −80 °C for subsequent experiments.

### 2.4 SDS–PAGE analysis

The protein quality and purity were evaluated by SDS–PAGE under denaturing conditions after quantification using a Qubit fluorometer and further confirmed by Bicinchoninic Acid Protein Assay (Sigma-Aldrich, St. Louis, MS, USA). Protein samples were mixed with Laemmli loading buffer containing β-mercaptoethanol, heated at 95 °C for 5 min, and separated on a 12% polyacrylamide gel. The proteins were visualized by Coomassie Brilliant Blue staining. Apparent molecular weights were estimated by comparison with a prestained protein molecular weight marker (PageRuler Prestained Protein Ladder, 10–180 kDa; Thermo Scientific, Lithuania).

### 2.5 Endotoxin quantification

Endotoxin level in the purified recombinant LieIF protein was assessed using a chromogenic Limulus Amebocyte Lysate assay (Pierce™ LAL Chromogenic Endotoxin Quantitation Kit, Thermo Scientific, USA), based on the generation of a chromogenic signal in the presence of endotoxins. A standard curve prepared with *E. coli* endotoxin was used to quantify endotoxin levels in protein samples. Absorbance was measured at 405 nm using a microplate reader (Multiskan™ GO, Thermo Scientific, Finland).

### 2.6 Neutrophil isolation and purity assessment

Polymorphonuclear neutrophils (PMNs) were isolated from peripheral blood of healthy adult volunteers donors using density centrifugation through Ficoll-Hypaque (GE Healthcare, Sweden), followed by dextran sedimentation, as previously described (25). Cell viability was assessed with 0.4% Trypan Blue (Sigma-Aldrich, St. Louis, MS, USA) and exceeded 99% in all preparations. The purity of the neutrophil preparations was analyzed immediately after their isolation (T0) and after 24 h of culture by flow cytometry. Cells were stained with fluorochrome-conjugated anti-human CD16-PerCP-Cy5.5 and anti-human CD14-FITC monoclonal antibodies (BD Biosciences, San Jose, CA, USA) according to the manufacturer’s instructions. After exclusion of debris and doublets, live cells were analyzed for CD14 and CD16 expression. Neutrophils were identified as CD16+CD14- cells, whereas contaminating monocytes were identified as CD14+ cells. Data were acquired using a BD FACSCanto II flow cytometer (BD Biosciences, San Jose, CA, USA) and analyzed with BD FACSDiva software.

### 2.7 Neutrophil stimulation and cell viability assay

To evaluate the effect of recombinant LieIF protein on neutrophil viability, we performed an MTT (Methylthiazolyldiphenyl-tetrazolium bromide) assay, as previously described (25, 26). Briefly, neutrophils were seeded at a density of 2 × 10^5^ cells per well in 96-well plates containing complete RPMI-1640/Glutamax medium containing penicillin (100 U/mL) and streptomycin (100 µg/mL) supplemented with 5% Fetal Bovine Serum (FBS), and incubated with increasing concentrations of recombinant LieIF protein (0, 0.31, 0.62, 1.25, 2.5 and 5 µg/mL) for 18 h. At the end of the incubation, 20 µL of 5 mg/mL MTT solution (Sigma-Aldrich, St. Louis, MS, USA) was added to each well, where viable cells reduced MTT to formazan crystals via mitochondrial dehydrogenases over 4 h at 37 °C in a humidified atmosphere containing 5% CO_2_. Formazan crystals were then dissolved by adding 150 µL of dimethyl sulfoxide (DMSO; Sigma-Aldrich, St. Louis, MS, USA). Absorbance was measured at 570 nm using a microplate reader (Labsystems MULTISCAN, Thermo Fisher Scientific, Waltham, MA, USA). Data were collected from four donors and the percentage of cells viability was calculated as follows:


$$Cell\;Viability\;(\%)=\frac{OD\;LeIF\;stimulated\;PMNs\;-\;OD\;blank}{OD\;unstimulated\;PMNs\;-\;OD\;blank}\;X\;100$$

### 2.8 Cell cytotoxicity assay (LDH)

The cytotoxicity of the recombinant LieIF protein on human neutrophils was assessed by measuring lactate dehydrogenase (LDH) release using the LDH Cytotoxicity Detection Kit (Roche Boehringer Mannheim, Rueil-Malmaison, Hauts-de-Seine, France) as described previously (26, 27). LDH is a cytosolic enzyme rapidly released into the culture supernatant upon cell membrane damage. Briefly, 2 × 10^5^ neutrophils per well were seeded in 96-well plates and incubated with increasing concentrations of LieIF (0, 0.31, 0.62, 1.25, 2.5 and 5 µg/mL) for 18 h. Cells treated with 1% Triton X-100 were used as positive controls for maximum LDH release. After 18 h of incubation, culture supernatants were collected and incubated for 30 minutes at 37 °C, 5% CO_2_ with the reaction substrate provided in the Cytotoxicity Detection Kit LDH (Roche Boehringer Mannheim, Rueil-Malmaison, Hauts-de-Seine, France). Extracellular LDH activity was measured spectrophotometrically at 492 nm using (Labsystems MULTISCAN, Thermo Fisher Scientific, Waltham, MA, USA). Data were collected from four donors performed in duplicate and the percentage of cells cytotoxicity was calculated as follows:


$$Cell\;Cytotoxicity\;(\%)=\frac{OD\;LeIF\;stimulated\;PMNs\;-\;OD\;unstimulated\;PMNs}{OD\;1\%\;Triton\;X100\;stimulated\;PMNs\;-\;OD\;unstimulated\;PMNs}\;X\;100$$

### 2.9 Measurement of reactive oxygen species production

Intracellular ROS levels were measured using the cell-permeable fluorogenic probe CM-H2DCFDA (Thermo Fisher, US). This non-fluorescent probe passively diffuses into cells and is deacetylated by intracellular esterases to 2′,7′-dichlorodihydrofluorescein (DCF-H2), which is rapidly oxidized to highly fluorescent DCF in the presence of Reactive Oxygen Species. Briefly, neutrophils were incubated with or without LieIF (2.5 µg/mL) for 2 h. Cells were then washed with PBS, resuspended in RPMI medium without phenol red, and incubated with 10 µM CM-H2DCFDA at 37 °C under 5% CO_2_ for 30 min in the dark. Fluorescence intensity was measured at an excitation/emission of 492/517 nm using a Varioskan Flash multimode microplate reader (Thermo Fisher, USA). Data are presented as mean ± SD from four donors.

### 2.10 Measurement of neutrophil elastase activity

Neutrophil elastase (NE) activity was assessed as previously described (25). Briefly, unstimulated and LieIF-stimulated neutrophils were incubated at 37 °C under 5% CO_2_ for 15 min, 30 min, 1 h, 2 h, 4 h, 6 h, 18 h, and 24 h. Culture supernatants were then collected by centrifugation at 200 × g for 10 min and incubated with 1 mM N-methoxysuccinyl-Ala-Ala-Pro-Val p-nitroanilide (Sigma, USA) to detect NE activity. After 3 h of incubation at 37 °C, the absorbance of cleaved p-nitroanilide was measured spectrophotometrically at 405 nm using a MULTISCAN GO microplate reader (Labsystems MULTISCAN, Thermo Fisher Scientific, Waltham, MA, USA). Data are presented as mean ± SD from four donors in technical replicates.

### 2.11 Measurement of myeloperoxidase activity

MPO activity in neutrophil supernatants was determined as previously described (25). Briefly, 100 µL of supernatants from unstimulated or LieIF-stimulated neutrophils (2.5 µg/mL) were collected at 15 min, 30 min, 1 h, 2 h, 4 h, 6 h, 18 h, and 24 h. An equal volume of substrate cocktail containing o-Dianisidine and H_2_O_2_ was then added. After 10 min of incubation at room temperature, the absorbance of oxidized o-Dianisidine was measured at 450 nm using the MULTISCAN GO microplate reader (Labsystems MULTISCAN, Thermo Fisher Scientific, Waltham, MA, USA). Data are presented as mean ± SD from four donors in technical replicates.

### 2.12 Measurement of neutrophils apoptosis

Cell apoptosis assay was assessed using the PE-Annexin V/7-amino-actinomycin D (7-AAD) apoptosis detection kit (BD Biosciences, San Jose, CA, USA) according to the manufacturer’s instruction. Briefly, 1 x 10^6^ neutrophils were incubated in the presence or absence of the recombinant LieIF protein (2.5 µg/mL) for 18h. PMNs were then washed with cold PBS and stained with 5 µL of Annexin V-PE/7-AAD for 15 min. Then, the stained cells were re-suspended in 100 µL of binding Buffer (1X) and were analyzed with flow cytometry (FACS Canto II, BD Biosciences, San Jose, CA, USA). Cell death was quantitatively evaluated by measuring the proportion of early (Annexin V-PE (+)/7-AAD (-)) and late (Annexin V-PE (+)/7-AAD (+)) apoptotic cells compared to viable (Annexin V-PE (-)/7-AAD (-)) and necrotic (Annexin V-PE (-)/7-AAD (+)) cells. Data are presented as mean ± SD values of the different cell populations from four donors.

### 2.13 Cytokines measurement

Human neutrophils were incubated in the presence or absence of recombinant LieIF protein (2.5 µg/mL). Cell culture supernatants were collected after 15 min, 30 min, 1 h, 2 h, 4 h, 6 h, 18 h, 24 h of treatment and then centrifuged at 10,000 rpm for 5 min to remove cellular debris. For TGF-β1 quantification only, culture supernatants were subjected to an acid activation step according to the manufacturer’s instructions. Briefly, samples were acidified with 1 N HCl and incubated for 60 min at 4 °C, followed by neutralization with 1 N NaOH prior to ELISA, allowing quantification of total TGF-β1 (latent and active forms). Cytokine levels were quantified using commercially available human ELISA kits (BD Biosciences, San Jose, CA, USA), according to the manufacturer’s instructions. Briefly, 96-well plates were coated overnight at 4 °C with purified anti-human capture antibodies diluted in carbonate-bicarbonate buffer (pH 9.5). After washing and blocking with PBS containing 10% FBS, samples or recombinant cytokine standards were added and incubated for 2 h at room temperature. Following extensive washing, biotinylated detection antibodies and streptavidin–horseradish peroxidase conjugate were added. The enzymatic reaction was developed using tetramethylbenzidine (TMB) substrate and stopped with sulfuric acid (2N). Absorbance was measured at 405 nm using a microplate reader (MULTISCAN GO, Thermo Scientific, Finland). Cytokine concentrations were calculated from standard curves and expressed as mean ± SD from four donors in technical replicates.

### 2.14 Western blot analysis

Control and LieIF-treated cells were analyzed at different time points and conditions. Cells were lysed in Laemmli sample buffer, and total protein concentrations were determined using the Bicinchoninic acid (BCA) protein assay kit (Sigma, USA). Equal amounts of protein (30 µg) were separated by SDS–polyacrylamide gel electrophoresis on 15% gels and transferred onto polyvinylidene difluoride (PVDF) membranes (Immobilon-P, Millipore). Following protein transfer, membranes were stained with Amido Black to assess overall protein loading and transfer efficiency. Membranes were then blocked with 5% non-fat dry milk in phosphate-buffered saline (PBS) for 1 h at room temperature and incubated overnight at 4 °C with the appropriate primary antibodies against: phospho-AKT (Ser473), total AKT, phospho-ERK1/2 (Thr202/Tyr204), total ERK1/2, p38 (Thr180/Tyr182), total p38, phospho-SAPK/JNK (Thr183/Tyr185) and total JNK1 (Cell Signaling Technology, Danvers, MA, USA), all diluted 1:1000. After washing, membranes were incubated with horseradish peroxidase-conjugated secondary antibodies (anti-mouse IgG or anti-rabbit IgG; 1:5000, Promega) for 2 h at room temperature. RAW 264.7 cells were stimulated with LPS (1 µg/mL) and used as positive controls for the detection of phosphorylated p38 MAPK, ERK1/2, JNK, and AKT by Western blot analysis. Immunoreactive bands were detected and visualized using a ChemiDoc MP Imaging System (Bio-Rad, Hercules, CA, USA) after incubation with enhanced chemiluminescence (ECL) detection system (Pierce, Rockford, USA).

### 2.15 Pharmacological inhibition studies

The involvement of specific signaling kinases in LieIF activity was investigated after PMNs pretreatments for 1 h with selective pharmacological inhibitors targeting p38 MAPK (SB203580, 10 µM), MEK/ERK (PD184352, 10 µM), JNK (SP600125, 10 µM) or PI3K/AKT (wortmannin, 100 nM), prior to their stimulation with LieIF (2.5 µg/mL) at indicated time. At the end of each incubation period, culture supernatants in both conditions (presence and absence of pharmacological inhibitors) were collected, centrifuged to remove cellular debris and stored at −80 °C until cytokine measurements using ELISA assay according to the manufacturers’ instructions. Corresponding cell pellets were harvested, lysed in Laemmli buffer and stored at −80 °C for subsequent Western blot analysis as described in section 2.14.

### 2.16 Statistical assessment

Graphs were generated using GraphPad Prism version 7.0 (GraphPad Software, San Diego, CA, USA). Data were shown as mean values ± standard deviation (SD). The significance of the results was calculated using the student t-test and differences between samples were considered significant when the *p*-value was < 0.05. On the figure, * (*p* < 0.05), indicates statistically significant differences at the indicated *p* values.

## 3 Results

### 3.1 Expression of the recombinant LieIF protein and purity characterization of isolated human polymorphonuclear neutrophils

**Figure 1:**
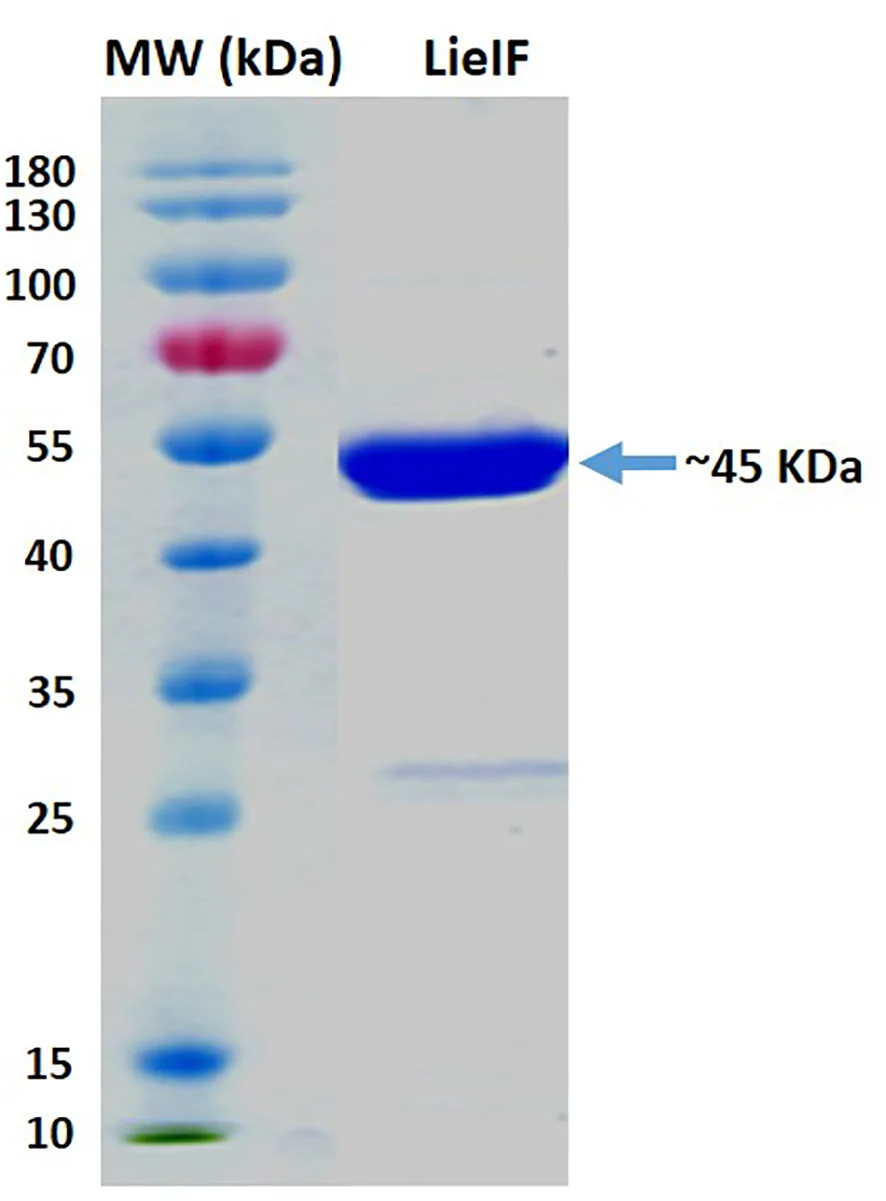
SDS-PAGE analysis of purified recombinant LieIF protein. Purified recombinant LieIF protein was analyzed by 12% SDS–PAGE under denaturing conditions and visualized by Coomassie Brilliant Blue staining. Lane 1: prestained protein molecular weight marker (PageRuler Prestained Protein Ladder, 10–180 kDa; Thermo Scientific). Lane 2: purified recombinant LieIF protein (1.5 µg). A major band with an apparent molecular weight of approximately 45.3 kDa was observed, consistent with the predicted size of LieIF protein.

The recombinant His-tagged LieIF was successfully produced and purified with a yield of 8 mg/L. Protein quality and molecular weight were assessed by SDS–PAGE on a 12% polyacrylamide gel. The analysis showed a major band with an apparent molecular mass of approximately 45.3 kDa, consistent with the expected size of the recombinant LieIF protein (**Figure 1**). Endotoxin contamination of the purified recombinant LieIF was evaluated using a chromogenic LAL assay. The measured endotoxin level was 1.10 EU/mL, corresponding to 0.88 EU per mg of protein, which is well below the maximum endotoxin threshold considered acceptable for *in vitro* experiments (5 EU/mg of protein).

Purified neutrophils were analyzed after their staining with monoclonal antibodies against CD16 and CD14. Flow cytometric analysis confirmed the high purity of the neutrophil preparations (CD16+CD14- cells). CD14 positive cells represented only 3.0% of the cell population immediately after isolation and declined to 1.2% after 24 h of culture, demonstrating negligible monocyte contamination throughout the study (**Supplementary Figure 1**).

### 3.2 Recombinant LieIF protein does not affect neutrophil viability or cell membrane integrity

**Figure 2:**
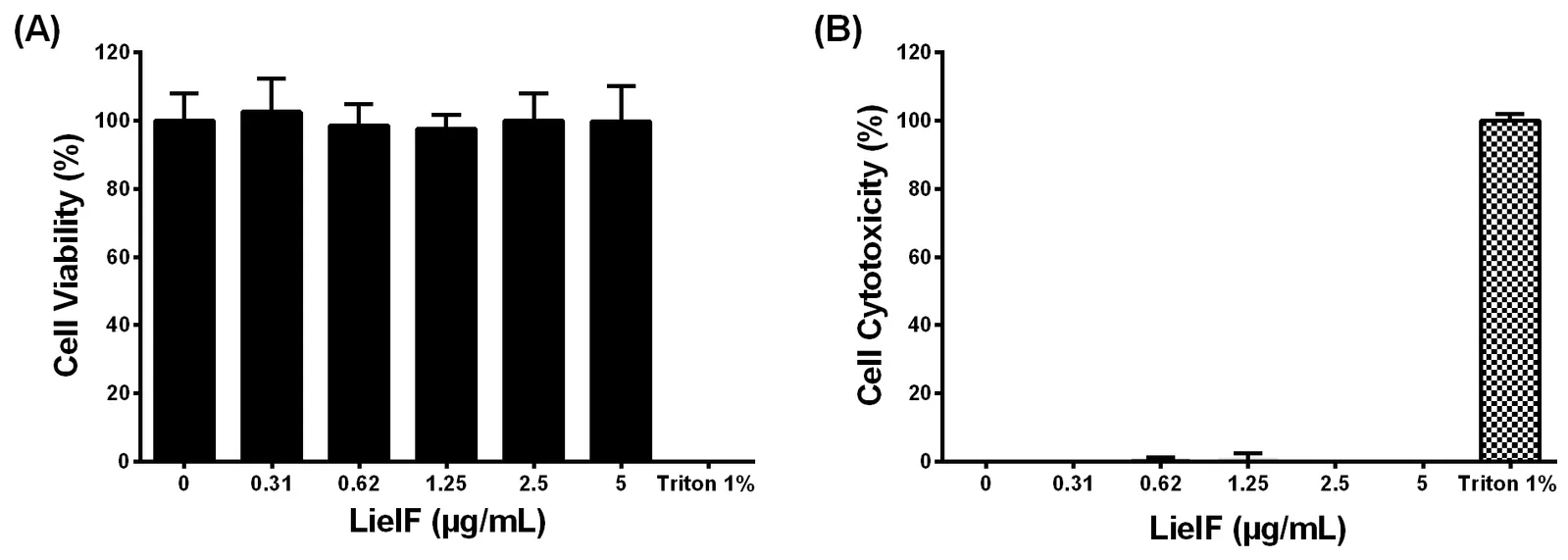
Effect of recombinant LieIF protein on human neutrophil viability or cell membrane integrity. Human neutrophils were cultured in 96-well plates and stimulated with increasing concentrations of recombinant LieIF (0, 0.31, 0.62, 1.25, 2.5 and 5 µg/mL) for 18 h. **(A)** Neutrophil viability was assessed using the MTT assay. **(B)** Cytotoxicity was evaluated by measuring lactate dehydrogenase (LDH) release in culture supernatants. Results are expressed as a percentage of maximal LDH release induced by 1% Triton X-100. Data of this image are presented as mean ± standard deviation (SD) of four donors performed in technical duplicates.

The effect of recombinant LieIF protein on human neutrophil viability was evaluated using the MTT assay after 18 h of incubation. As shown in **Figure 2A**, treatment with increasing concentrations of LieIF (0.31–5 µg/mL) did not alter neutrophil viability compared to untreated cells. Viability values remained comparable across all conditions, indicating that LieIF does not affect neutrophil metabolic activity. In parallel, cell membrane integrity was assessed by measuring LDH release using the LDH cytotoxicity assay kit as described previously above. Compared to the positive control (1% Triton X-100) that induces 100% of cytotoxicity and maximal LDH release, LieIF treatment at the tested concentrations did not affect LDH levels that remain similar to those of unstimulated neutrophils (**Figure 2B**). Taken together, these results indicated that recombinant LieIF protein does not compromise neutrophil viability nor induce cytotoxic effects under the experimental conditions used. Thus, to ensure that all subsequent studies exploring neutrophil response to recombinant LieIF protein reflect specific biological activities, a non-cytotoxic intermediate concentration of 2.5 µg/mL was selected for further experiments.

### 3.3 Recombinant LieIF protein stimulates reactive oxygen species production in human neutrophils

**Figure 3:**
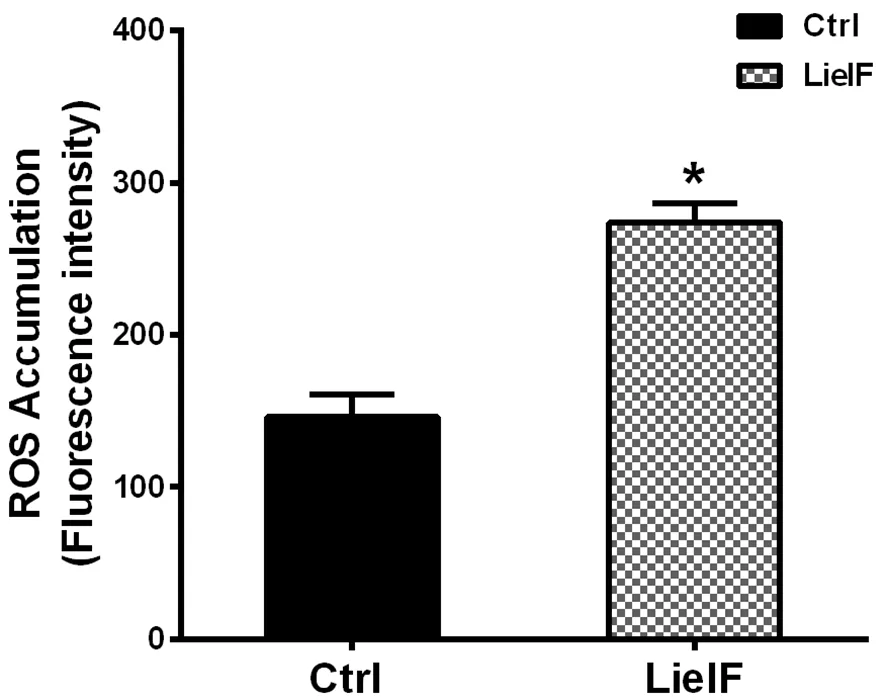
Measurement of reactive oxygen species (ROS) production. Human neutrophils were incubated with or without recombinant LieIF (2.5 µg/mL) for 2 h. Cells were then washed and incubated with 10 µM CM-H2DCFDA (Life Technologies, Oregon, USA) for 30 min at 37 °C under 5% CO_2_ in the dark. Intracellular ROS levels were quantified by measuring fluorescence at 492/517 nm (excitation/emission) using a multimode microplate reader. Data represent the mean ± SD from four donors. Statistical differences between unstimulated (Ctrl) and LieIF-stimulated cells were determined using Student’s t-test (*p* < 0.05).

Reactive oxygen species play a central role in neutrophil activation and antimicrobial host defense. To assess the effect of LieIF on ROS generation, human neutrophils were incubated with or without LieIF (2.5 µg/mL) for 2 h, and intracellular ROS levels were measured using the CM-H2DCFDA probe. The 2 h time point was selected based on the established kinetics of neutrophil oxidative burst, which typically peaks within the first hours of stimulation (28). As shown in **Figure 3**, unstimulated neutrophils displayed low basal ROS production, whereas LieIF stimulation induced a clear increase in intracellular ROS levels (*p* < 0.05). In neutrophils, ROS production is mainly associated with activation of the NADPH oxidase complex, although additional sources such as mitochondria and granule-associated enzymes may also contribute to intracellular ROS generation. These findings indicated that LieIF promotes a robust oxidative response in human neutrophils.

### 3.4 Recombinant LieIF protein induces neutrophil degranulation

Release of neutrophil granules contents contributes to the elimination of invading pathogens. Given the role of ROS in neutrophil activation, we next investigated whether recombinant LieIF protein could promote granule release.

**Figure 4:**
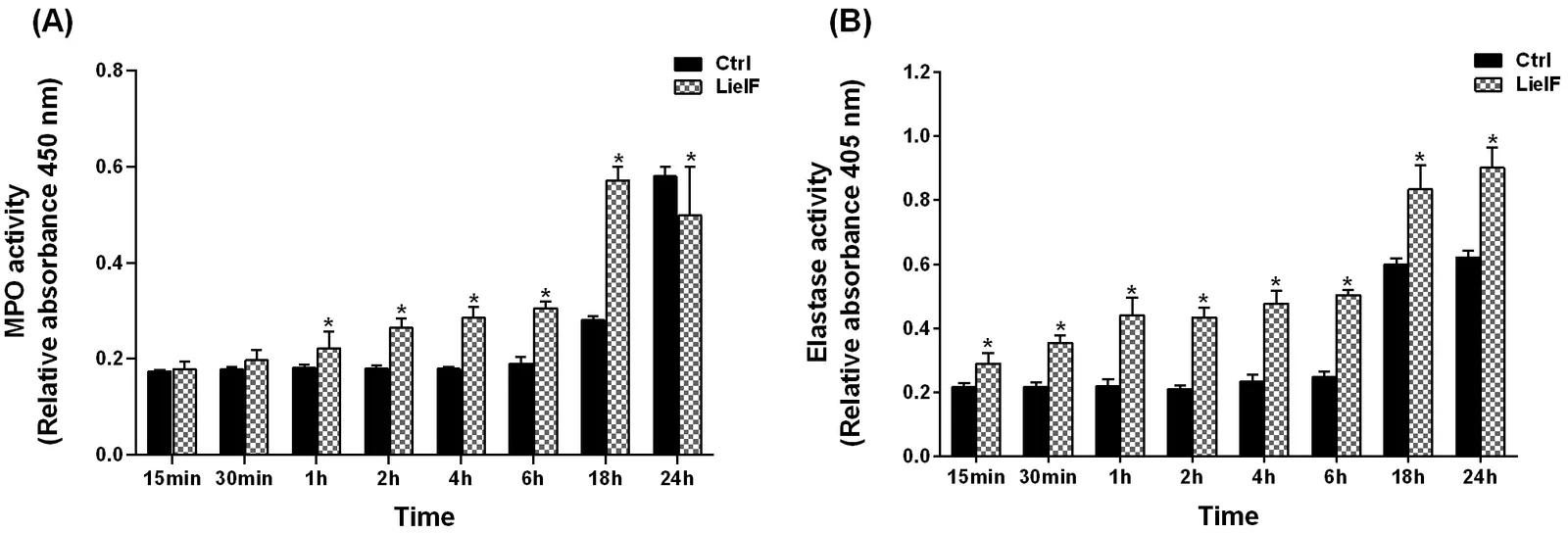
Kinetics of MPO and elastase release in neutrophils stimulated with recombinant LieIF protein. Human neutrophils were incubated with or without recombinant LieIF protein (2.5 µg/mL). Culture supernatants were collected at 15 min, 30 min, 1 h, 2 h, 4 h, 6 h, 18 h, and 24 h and analyzed for enzyme release. **(A)** MPO activity was measured using an enzyme substrate reaction with o-Dianisidine and H2O2. **(B)** Neutrophil elastase activity was quantified using the specific synthetic peptide substrate N-succinyl-Ala-Ala-Pro-Val p-nitroanilide. Data represent mean ± SD from for donors. Statistical differences between unstimulated (Ctrl) and LieIF-stimulated cells were determined using Student’s t-test (*p* < 0.05).

Thus, we examined the release of myeloperoxidase (MPO) and neutrophil elastase (NE) by un-stimulated and LieIF- stimulated cells. As shown in **Figure 4**, both enzymes were released in a time-dependent manner following LieIF stimulation. MPO release started at 1 h post treatment and peaked at 18 h and subsequently declined at 24 h (**Figure 4A**), whereas neutrophil elastase activity started at 15 min post treatment, reached its maximum at 18 h and remained elevated at 24 h of incubation (**Figure 4B**).

Taken together, these results demonstrated that the recombinant LieIF protein induces the degranulation of human neutrophils reflected by the release of MPO and elastase.

### 3.5 Recombinant LieIF protein increases late apoptosis in human neutrophils

**Figure 5:**
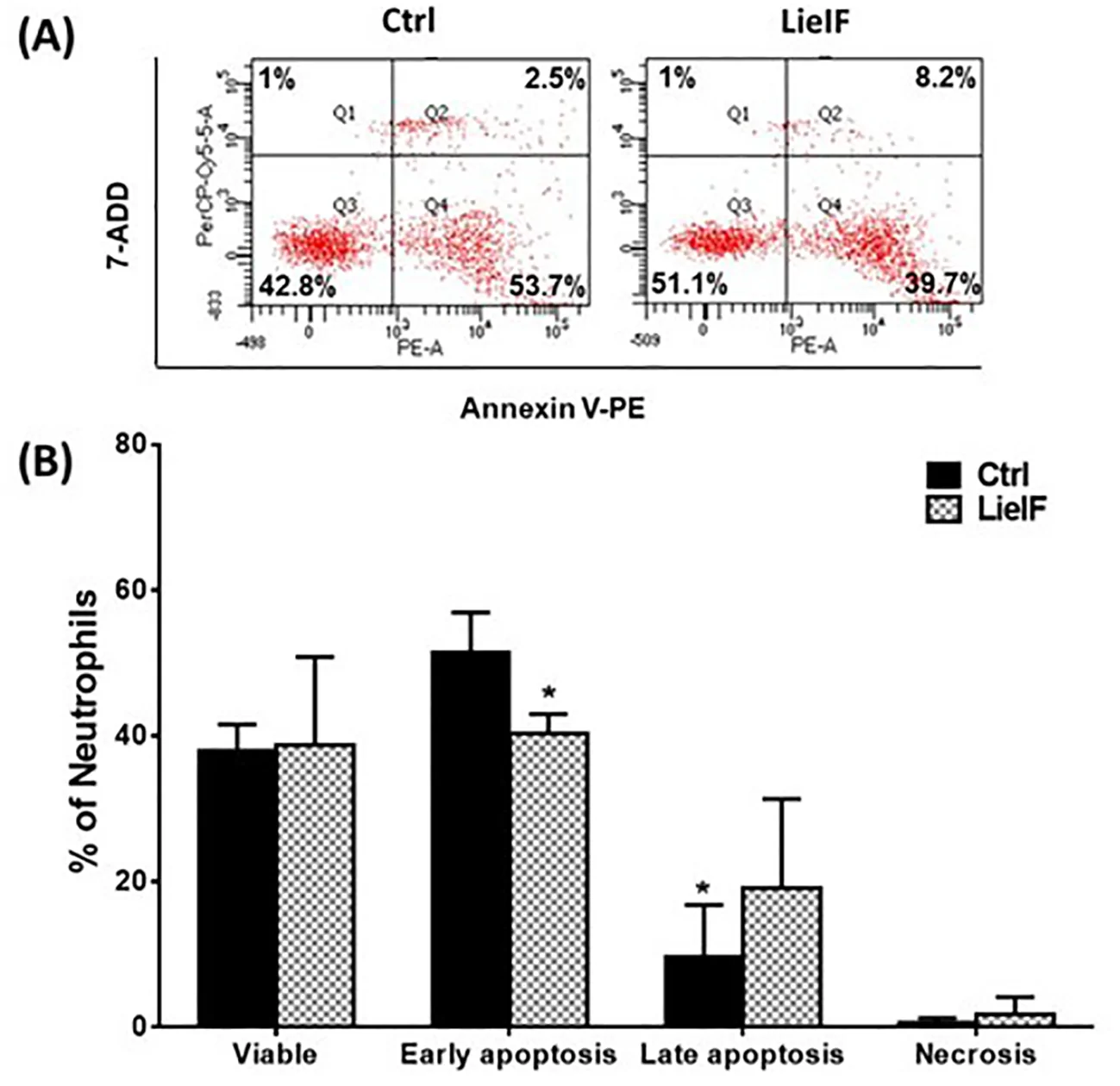
Assessment of neutrophil apoptosis by Annexin V/7-AAD flow cytometry. Human neutrophils isolated from four donors were incubated in the presence or absence of LieIF (2.5 µg/mL) for 18 h and then labeled with PE–Annexin V and 7-amino-actinomycin D (7-AAD) for flow cytometry analysis. **(A)** Representative dot plots from one donor showing viable (Annexin V^-^/7-AAD^-^), early apoptotic (Annexin V^+^/7-AAD^-^), late apoptotic (Annexin V^+^/7-AAD^+^), and necrotic (Annexin V^-^/7-AAD^+^) neutrophil populations. **(B)** The percentage of each cell population from four independent donors are presented as the mean ± SD. Statistical differences between unstimulated (Ctrl) and LieIF-stimulated cells were assessed using Student’s t-test (*p* < 0.05).

Neutrophils are short-lived cells that undergo spontaneous apoptosis, limiting their antimicrobial functions and weakening the early innate immune response against invading pathogens. Sustaining neutrophil survival is therefore essential, particularly during *Leishmania* infection where early innate responses shape disease outcome (4, 29). To assess the effect of the recombinant LieIF protein on human neutrophil apoptosis, cells were incubated in the presence or absence of LieIF (2.5 µg/mL) for 18 h. This time point was selected as it corresponds to the period of maximal spontaneous neutrophil apoptosis under standard culture conditions, representing the most sensitive window for detecting anti-apoptotic effects. The percentage of apoptotic neutrophils was determined using the PE-Annexin V/7-amino-actinomycin D (7-AAD) apoptosis detection kit. As shown in **Figure 5**, LieIF-treated neutrophils displayed a slight reduction in the proportion of early apoptotic cells (40%) compared to untreated control (51%) that was concomitant to significant increase in the percentage of late apoptotic cells by approximately 2.5 fold. However, when early and late apoptotic populations were considered together, the proportion of Annexin V-positive cells remained comparable between control and LieIF-treated conditions. The percentage of necrotic cells remained very low (<1.8%) in both conditions. These findings suggest that recombinant LieIF protein promotes late apoptosis in human neutrophils rather than prolonging their survival.

### 3.6 Recombinant LieIF protein stimulates cytokines production by human neutrophils

**Figure 6:**
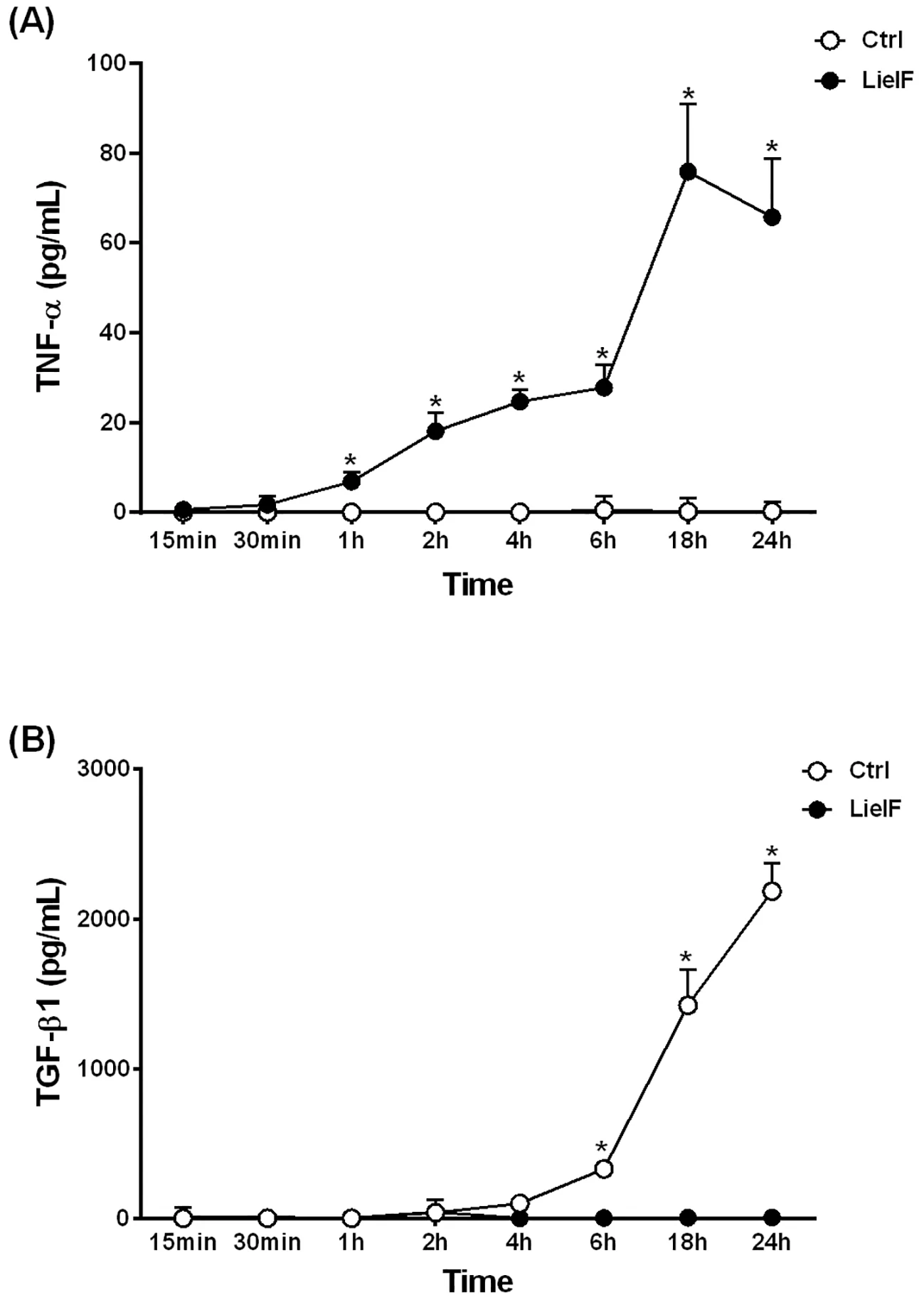
Kinetics of TNF-α and TGF-β1 production by human neutrophils in response to LieIF. Human neutrophils were incubated with or without recombinant LieIF protein (2.5 µg/mL). Cell-free supernatants were collected after 15 min, 30 min, 1 h, 2 h, 4 h, 6 h, 18 h, and 24 h of incubation. TNF-α **(A)** and TGF-β1 **(B)** concentrations were quantified by ELISA. Results are expressed as mean ± SD from four donors. Statistical comparisons between unstimulated and LieIF-stimulated neutrophils were performed using Student’s *t*-test. *p* < 0.05 was considered statistically significant.

Neutrophils are key regulators of the innate immune response through the secretion of cytokines (30). In *Leishmania* models, TNF-α promotes parasite killing while TGF-β- mediated immunosuppressive effect favors parasite survival and disease progression (31, 32). To investigate the effect of recombinant LieIF on cytokine secretion by human neutrophils, we first analyzed the kinetics of TNF-α and TGF-β1 production using ELISA at multiple time points. ELISA analysis revealed distinct secretion patterns for TNF-α and TGF-β1 (**Figure 6**). TNF-α was not detectable in unstimulated neutrophils, whereas stimulation with LieIF induced a clear time-dependent increase in TNF-α secretion. TNF-α became detectable after 1 h of stimulation, increased progressively over time, and reached maximal levels at 18 h, followed by a slight decrease at 24 h (**Figure 6A**).

In contrast for TGF-β1, unstimulated neutrophils exhibited constitutive production of this cytokine with detectable basal levels observed from 6 h onward, which further increased and peaked at 24 h in control cells, indicating spontaneous secretion of this anti-inflammatory cytokine over time. Notably, LieIF stimulation markedly reduced TGF-β1 production, maintaining cytokine levels close to baseline at different analyzed time points (**Figure 6B**). These results demonstrate that recombinant LieIF protein promoted the secretion of the pro-inflammatory TNF-α cytokine while inhibited that of the anti-inflammatory cytokine TGF-β1 suggesting a differentially regulation of both cytokines production by human neutrophils.

### 3.7 Recombinant LieIF protein induces selective activation of the p38 MAPK pathway in human neutrophils

**Figure 7:**
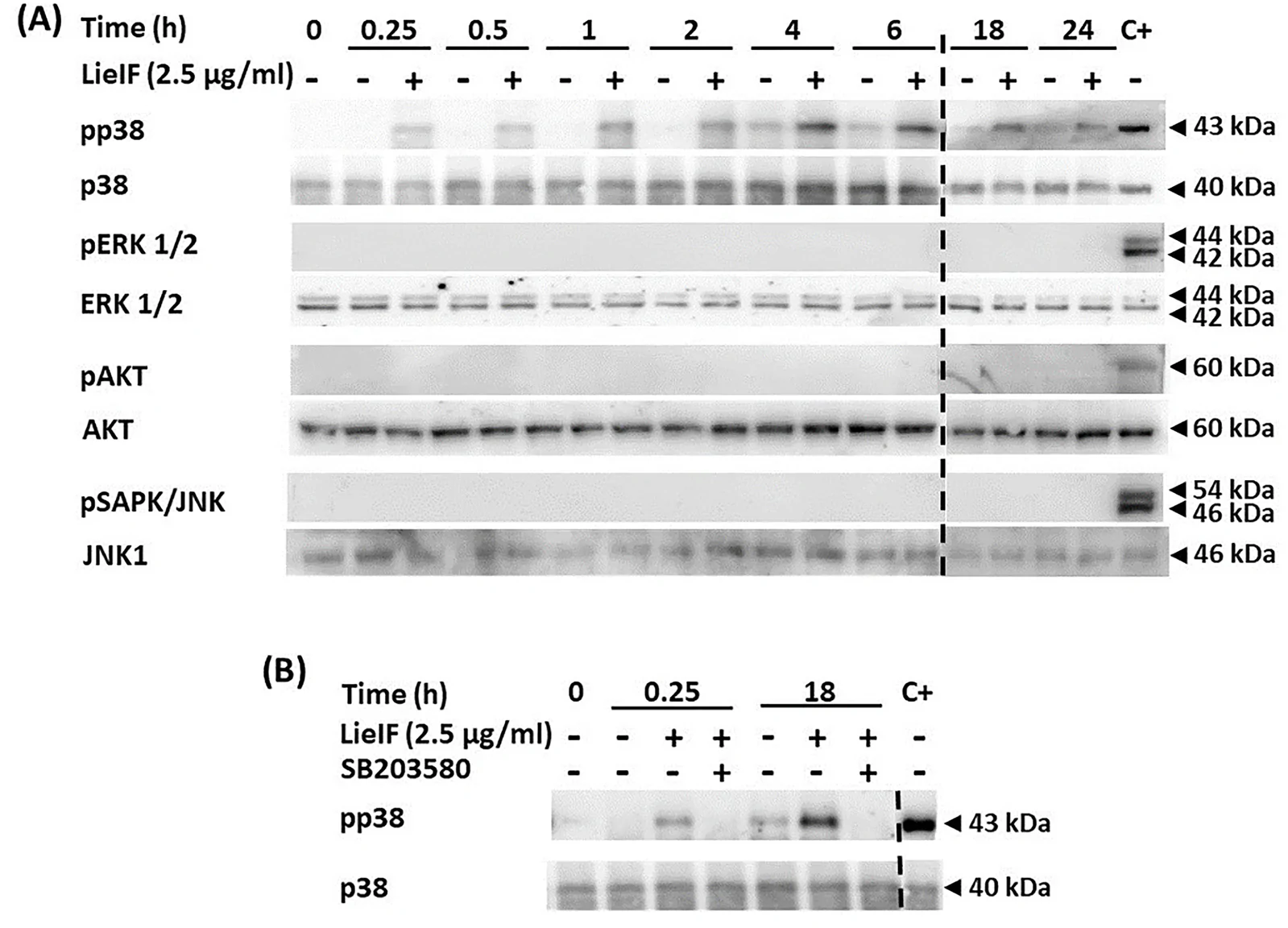
Western blot analysis of MAPK (p38, ERK1/2 and JNK) and PI3K/activation in LieIF-stimulated human neutrophils. Human neutrophils were incubated in the absence or presence of recombinant LieIF protein (2.5 µg/mL) for the indicated time points. Total cellular proteins (30 µg per lane) were resolved by SDS–PAGE and analyzed by Western blotting. **(A)** Time-course analysis of p38 MAPK, AKT, ERK1/2, and JNK phosphorylation in unstimulated and LieIF-stimulated neutrophils at the indicated time points. Corresponding total protein levels (p38 MAPK, ERK1/2, AKT and JNK) were analyzed to confirm equal protein loading and to normalize phosphorylated protein signals. **(B)** Effect of signaling pathway inhibitors on LieIF-induced p38 MAPK phosphorylation. Neutrophils were pretreated with SB203580 (p38 MAPK inhibitor) for 1h prior to LieIF stimulation. Phosphorylation of p38 MAPK was analyzed at early (0.25 h) and late (18 h) time points. LPS-stimulated RAW 264.7 cells were used as a positive control.

Mitogen-activated protein kinase (MAPK) and PI3K pathways play a central role in transducing extracellular signals into transcriptional and functional responses in immune cells. To determine whether recombinant LieIF protein activates these intracellular signaling cascades in human neutrophils, the phosphorylation status of key kinases involved in the MAPK and PI3K pathways was analyzed by Western blotting. Unstimulated and LieIF-stimulated neutrophils were analyzed at early and late time points ranging from 0.25 h to 24 h. As shown in **Figure 7A**, LieIF induced a rapid and sustained phosphorylation of p38 MAPK detectable at 0.25 h and persisting up to 24 h, indicating a strong and prolonged activation of this kinase. In contrast, no effect was observed on the phosphorylated forms of AKT, ERK1/2 and JNK kinases following LieIF stimulation, although phosphorylation of these kinases was observed in the LPS-stimulated RAW 264.7 cells used as positive controls (**Figure 7A**). To highlight equal protein loading across different conditions, a representative Amido Black staining of the PVDF membranes is shown (**Supplementary Figure 2**).

Since *Leishmania* infection can modulate host signaling pathways, we further investigated whether LieIF-induced p38 MAPK activation is maintained in the context of parasite infection. Human neutrophils were pretreated with LieIF and subsequently infected with *L. major* (EMPA-12). As shown in **Supplementary Figure 3**, *L. major* infection induced p38 MAPK phosphorylation, which was further enhanced in LieIF-pretreated infected neutrophils, whereas no effect on ERK1/2 phosphorylation was detected under these experimental conditions. This result demonstrates that LieIF-mediated activation of the p38 MAPK pathway is maintained during *Leishmania* infection under our experimental conditions, suggesting a potential role for this kinase during host–parasite interactions.

To further confirm the involvement of the p38 MAPK in LieIF-induced signaling, neutrophils were pretreated with pharmacological inhibitors targeting p38 kinase (SB203580) prior to LieIF stimulation. Western blot analysis showed that SB203580 reduced LieIF-induced p38 MAPK phosphorylation at both early (0.25 h) and late (18 h) time points, compared to cells treated with LieIF alone (**Figure 7B**) highlighting that the activation response induced by LieIF is mediated through the p38 MAPK pathway.

Taken together, these results suggest that recombinant LieIF protein induces a selective activation of the p38 MAPK pathway both in non-infected human neutrophils and in the context of *Leishmania* infection.

### 3.8 The p38 MAPK signaling regulates LieIF-induced cytokine production in human neutrophils

Given the selective activation of p38 MAPK by LieIF, we next examined the contribution of this intracellular signaling pathway to the LieIF-induced cytokine production by human neutrophils. Neutrophils were pretreated with a specific pharmacological inhibitor of p38 MAPK (SB203580) compared to inhibitors targeting other kinases such as MEK/ERK (PD184352), JNK (SP600125) or PI3K/AKT (wortmannin) prior to LieIF stimulation, and cytokine levels were quantified after 18 h of incubation.

**Figure 8:**
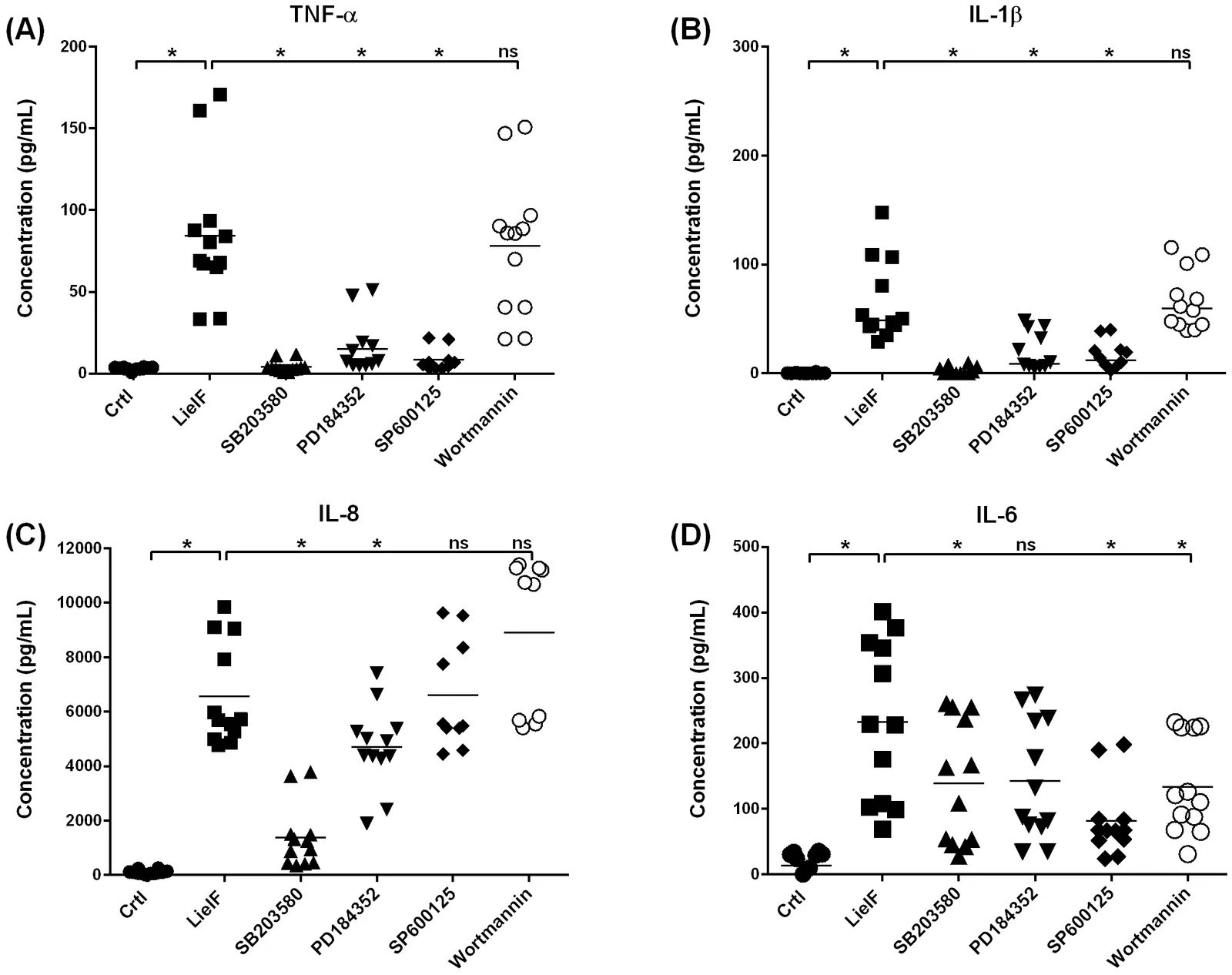
Quantification of cytokine secretion by human neutrophils in response to recombinant LieIF protein and signaling pathway inhibitorsHuman neutrophils were stimulated with the recombinant LieIF protein (2.5 µg/mL) for 18 h. To assess the involvement of intracellular signaling pathways, neutrophils were pretreated with pharmacological inhibitors of p38 MAPK (SB203580), MEK/ERK (PD184352), JNK (SP600125) or PI3K/AKT (wortmannin), for 1 h prior to LieIF stimulation. Supernatants were collected and levels of **(A)** TNF-α , **(B)** IL-1β, **(C)** IL-8, and **(D)** IL-6 were measured by ELISA. Data are expressed as mean ± SD of technical replicates from four donors. Statistical analyses were performed by comparing untreated versus LieIF-treated cells, and LieIF-treated cells versus LieIF-treated cells in the presence of each inhibitor. The p value < 0.05 was considered statistically significant

LieIF stimulation significantly induced the secretion of the pro-inflammatory cytokines TNF-α, IL-1β, IL-8, and IL-6 compared with untreated neutrophils, which did not produce detectable levels of these cytokines (**Figure 8**). In contrast, IL-10, IL-12p70, and IFN-γ were not detected in neutrophil supernatants, either in the presence or in absence of LieIF, indicating that these cytokines are not produced by human neutrophils under the experimental conditions used.

Pharmacological inhibition of p38 MAPK using SB203580 completely abolished LieIF-induced TNF-α and IL-1β secretions (**Figures 8A, B**) and strongly reduced IL-8 levels (**Figure 8C**), while partially decreasing IL-6 production (**Figure 8D**). Inhibition of MEK/ERK reduced TNF-α, IL-1β and IL-8 secretion, with no significant effect on IL-6. In contrast, blockade of the JNK pathway selectively reduced TNF-α, IL-1β and IL-6 production without affecting IL-8 levels, whereas inhibition of PI3K/AKT specifically impaired IL-6 secretion, with no significant effect on the other analyzed cytokines (**Figure 8**). Overall, these results indicated that LieIF-induced pro-inflammatory cytokine production in human neutrophils is mainly dependent on p38 MAPK signaling, while PI3K/AKT MAPK, MEK/ERK and JNK pathways contribute to cytokine regulation. Together, these findings identify p38 MAPK as the central signaling pathway that cooperate with other signaling cascades to control LieIF-induced inflammatory responses in human neutrophils.

## 4 Discussion

Neutrophils are among the first immune cells recruited to the site of *Leishmania* infection and are critical in shaping early host–parasite interactions. While their contribution to parasite clearance or persistence has been widely documented (4, 33), the mechanisms by which individual parasite-derived molecules regulate neutrophil effector functions remain poorly defined. In this study, we demonstrate that the *L. infantum* eukaryotic initiation factor 4A (LieIF), an excreted/secreted parasite protein, acts as a potent activator of human neutrophils, inducing a robust pro-inflammatory response through selective engagement of the p38 MAPK signaling pathway.

LieIF induced a marked increase in reactive oxygen species (ROS) production and triggered robust neutrophil degranulation, as reflected by the sustained release of myeloperoxidase (MPO) and neutrophil elastase (NE), two key components of azurophilic granules. These enzymes play a central role in antimicrobial defense, contributing to the degradation of pathogens within phagolysosomes. These effector mechanisms represent central antimicrobial strategies employed by neutrophils to control *Leishmania* infection (28, 34–36). Consistent with previous reports, ROS generation and granule release are well-established responses to whole parasites across multiple *Leishmania* species (25, 36–38). In contrast to the extensive literature on whole parasites, only a limited number of studies have investigated the effects of isolated *Leishmania*-derived molecules on neutrophil effector functions. This activation profile contrasts with the predominantly immunosuppressive properties described for most *Leishmania* surface molecules. For instance, lipophosphoglycan (LPG) dampens neutrophil ROS production to facilitate parasite survival (39), GP63 inhibits chemotaxis and oxidative burst (40), and *L. donovani* surface membrane acid phosphatases suppress the oxidative burst through enzymatic dephosphorylation (41). LieIF therefore represents a functionally distinct class of parasite-derived immunostimulatory factor, with an activation signature closely resembling that of ArtinM, a plant lectin that similarly induces ROS, degranulation, and pro-inflammatory cytokines while reducing TGF-β in human neutrophils (42).

In addition to functional neutrophil activation, LieIF modulated the spontaneous neutrophil cell death process by impacting the percentage of early and late apoptotic cells, without affecting their total proportion.

This effect may have complex and context-dependent implications in the setting of leishmaniases highlighting the well documented double-edged role of apoptosis in infectious disease. On one hand, modulation of apoptotic dynamics may influence the functional lifespan of neutrophils and the timing of their clearance, thereby potentially affecting antimicrobial responses and interactions with other immune cells (43, 44). On the other hand, altered apoptotic responses may also influence parasite fate, as *Leishmania* parasites can exploit neutrophil survival and apoptotic pathways to establish a transient intracellular niche and facilitate subsequent transfer to macrophages (45, 46). Therefore, whether LieIF-induced modulation of neutrophil apoptosis contributes to host protection or favors parasite persistence remains to be determined and will require further studies investigating parasite uptake, intracellular survival, and neutrophil–macrophage interactions. We have also previously shown that *Leishmania* species and strains similarly increased apoptosis but triggered differential cytokines release, and the rescued parasites from these infected cells presented different survival rates (25). It will be therefore relevant to also investigate in future studies the effect of LeIF on the infection outcomes according to the genetic background of *Leishmania* parasites and to elucidate further the mechanisms involved.

A major finding of this study is the selective modulation of neutrophil cytokine production by LieIF. We found that LieIF induced a time-dependent secretion of TNF-α while suppressing the anti-inflammatory cytokine TGF-β1, indicating a shift toward a pro-inflammatory profile. Notably, this cytokine balance is highly relevant in the context of leishmaniases, where elevated TGF-β levels are associated with susceptibility to *L. major* infection, whereas increased TNF production correlates with resistance (31, 32, 47). Comprehensive cytokine profiling further confirmed that LieIF strongly induces TNF-α, IL-1β, IL-6, and IL-8 key mediators of inflammation and leukocyte recruitment. These findings extend the well-characterized immunomodulatory properties of LieIF across multiple immune cell types. Our team and others have shown that recombinant LieIF protein promotes Th1 polarization, induces cytokine production by Human Peripheral Blood Mononuclear Cells (PBMC) and monocytes, drives dendritic cell maturation, and enhances macrophage microbicidal activity (17, 19–23). In addition, its ability to activate innate immune responses in lymphocyte-deficient models supports the involvement of pattern recognition mechanisms (48). Our group has further demonstrated that *in vivo* administration of recombinant LieIF protein promotes recruitment of neutrophils and monocytes, an activity comparable to the adjuvant alum (49). Together, these findings prompted us to investigate the underlying signaling pathways governing this pro-inflammatory response.

Given this pronounced pro-inflammatory cytokine profile, we next investigated the signaling pathways underlying LieIF-induced neutrophil activation. We found that recombinant LieIF protein selectively activated the p38 MAPK pathway in human neutrophils, without inducing phosphorylation of AKT, ERK1/2, or JNK. The p38 MAPK is a stress-activated serine/threonine kinase activated by dual phosphorylation of Thr180 and Tyr182 by the upstream kinases MKK3 and MKK6 (50). In neutrophils, p38 MAPK functions as a central regulator of cytokine production, degranulation, chemotaxis and survival (51–53). Importantly, LieIF-induced p38 MAPK activation was also maintained in the context of *L. major* infection and further enhanced in LieIF-pretreated neutrophils, while the phosphorylated form of ERK1/2 remained undetectable. This finding contrasts with previous reports showing that *Leishmania* infection can activate ERK1/2 in human neutrophils. This response was associated in some cases with delayed apoptosis (54) along with p38 and AKT activations under co-stimulatory conditions such as extracellular ATP (55). These different cellular outcomes may be explained by the tested parasite species/strains, the infection protocol, the nature of co-stimulatory signals and the time course, which suggested that the MAPK activation in human neutrophils upon *Leishmania* infection is highly context-dependent.

Pharmacological inhibition with SB203580 was then used to assess the functional contribution of p38 MAPK to cytokine production. SB203580 markedly reduced TNF-α and IL-1β secretions, strongly decreased IL-8, and partially reduced IL-6, supporting p38 as the major kinase involved in LieIF-induced cytokine production. In line with these findings, pharmacologic inhibition of p38 MAPK activation has been shown to broadly suppress neutrophil effector functions, including respiratory burst activity, exocytosis, chemotaxis, adhesion, IL-8 synthesis, and stress-induced apoptosis (52). Consistent with its established mechanism of action, SB203580 reduces p38 phosphorylation at Thr180 and Tyr182 residues and inhibits the kinase activity of p38 by occupying its ATP-binding pocket without interfering with upstream MKK3/6-mediated activation (52, 56). Beyond the selective activation of p38 without altering the phosphorylation status of ERK1/2, AKT or JNK, pharmacological inhibition of these kinases also reduced LieIF-induced cytokine secretion. This suggests that neutrophils maintain a basal, tonic activity of these pathways (57, 58), which interacts and supports p38-driven cytokine production, notably through convergence on shared downstream transcriptional regulators such as NF-κB and AP-1 (59, 60) rather than being independently triggered by LieIF. Indeed, the impact of these selective inhibitors on cytokines production may reflect complex intracellular crosstalk or their inherent off-target constraints in primary cells. Our data underscore that p38 MAPK serves as the central upstream, stimulus-responsive kinase in this response, with the contribution of ERK, AKT and JNK kinases by providing a necessary homeostatic framework for downstream effector functions. Although LieIF induced robust neutrophil degranulation, whether neutrophil-derived proteases released during this process can proteolytically process or degrade the recombinant protein remains unknown. Interestingly, neutrophil elastase and MPO release occurred early after stimulation, whereas LieIF-induced p38 MAPK activation and pro-inflammatory cytokine production persisted at later time points. Even if these observations do not exclude partial proteolytic processing of LieIF, they suggest that sufficient biological activity is maintained throughout the time course of the experiments. It is worth to note that future studies are warranted to assess whether neutrophil-derived proteases regulate the stability, bioavailability, and duration of LieIF activity.

The precise receptor responsible for LieIF recognition on neutrophils remains an important open question. TLR4 can be excluded as a candidate, as LieIF activated innate immune cells in TLR4-deficient C3H/HeJ mice (48). Whether TLR2, which recognizes multiple *Leishmania*-derived ligands and plays a key role in early innate responses to the parasite (61, 62), contributes to neutrophil sensing of LieIF remains to be formally investigated. Identifying the LieIF receptor(s) on neutrophils will be essential to fully elucidate the molecular basis of its immunostimulatory properties and to better understand its role in the innate immune response to *Leishmania*. Together, these findings position LieIF as a multifunctional immunomodulatory molecule capable of orchestrating complementary effector programs across innate immune cells.

From a translational perspective, the NH_2_-terminal domain of LieIF constitutes a component of the Leish-111f (also known as LEISH-F1), a polyprotein vaccine which has demonstrated protective efficacy in preclinical models and reached Phase II clinical evaluation (63–70). In parallel, our group has identified LieIF as a druggable target, with small-molecule inhibitors of its ATPase activity showing leishmanicidal effects against both promastigote and intracellular amastigote stages without detectable host cytotoxicity (71). Together along with the present findings, these data highlight the dual translational relevance of LieIF as both a neutrophil-activating innate immune stimulus and a validated therapeutic target, reinforcing the rationale for its continued investigation. Beyond the immunomodulatory effects highlighted in the present study, several questions remain open for future investigation. Whether LieIF influences *Leishmania* uptake by neutrophils, and how this may in turn affect parasite survival and subsequent transfer to macrophages, also remains to be determined. Future studies using neutrophil–parasite and neutrophil–infected macrophage co-culture models will help clarify the relevance of LieIF-induced neutrophil activation within the broader host–parasite interaction.

In conclusion, this study provides the first comprehensive characterization of LieIF-induced activation of primary human neutrophils, demonstrating that this conserved excreted/secreted parasite protein drives a coordinated pro-inflammatory program encompassing oxidative burst, degranulation, cytokine secretion, and prolonged survival through selective activation of the p38 MAPK pathway. The fact that a single secreted molecule is sufficient to recapitulate key features of the early neutrophil response to *Leishmania* underscores the immunoregulatory potency of LieIF at the host–parasite interface. These findings provide new insight into early host–parasite interactions and open avenues for targeting parasite-derived immunomodulators. Given its established role as a vaccine component and validated drug target, the present findings add a new and important dimension to the biology of this molecule, with direct implications for the rational design of novel anti-leishmanial therapeutic and vaccine strategies.

## Data Availability

The original contributions presented in the study are included in the article/**Supplementary Material**. Further inquiries can be directed to the corresponding author.
