# Supplementary material for: Leishmania infantum eukaryotic initiation factor 4A modulates in vitro functional activity of human neutrophils
Source: Front Immunol. 2026 Sep 1;17:1873341. doi: 10.3389/fimmu.2026.1873341 (PMC13574617; doi:10.3389/fimmu.2026.1873341)
Supplement: Supplementary file 1 [file DataSheet1.pdf]

Supplementary Material

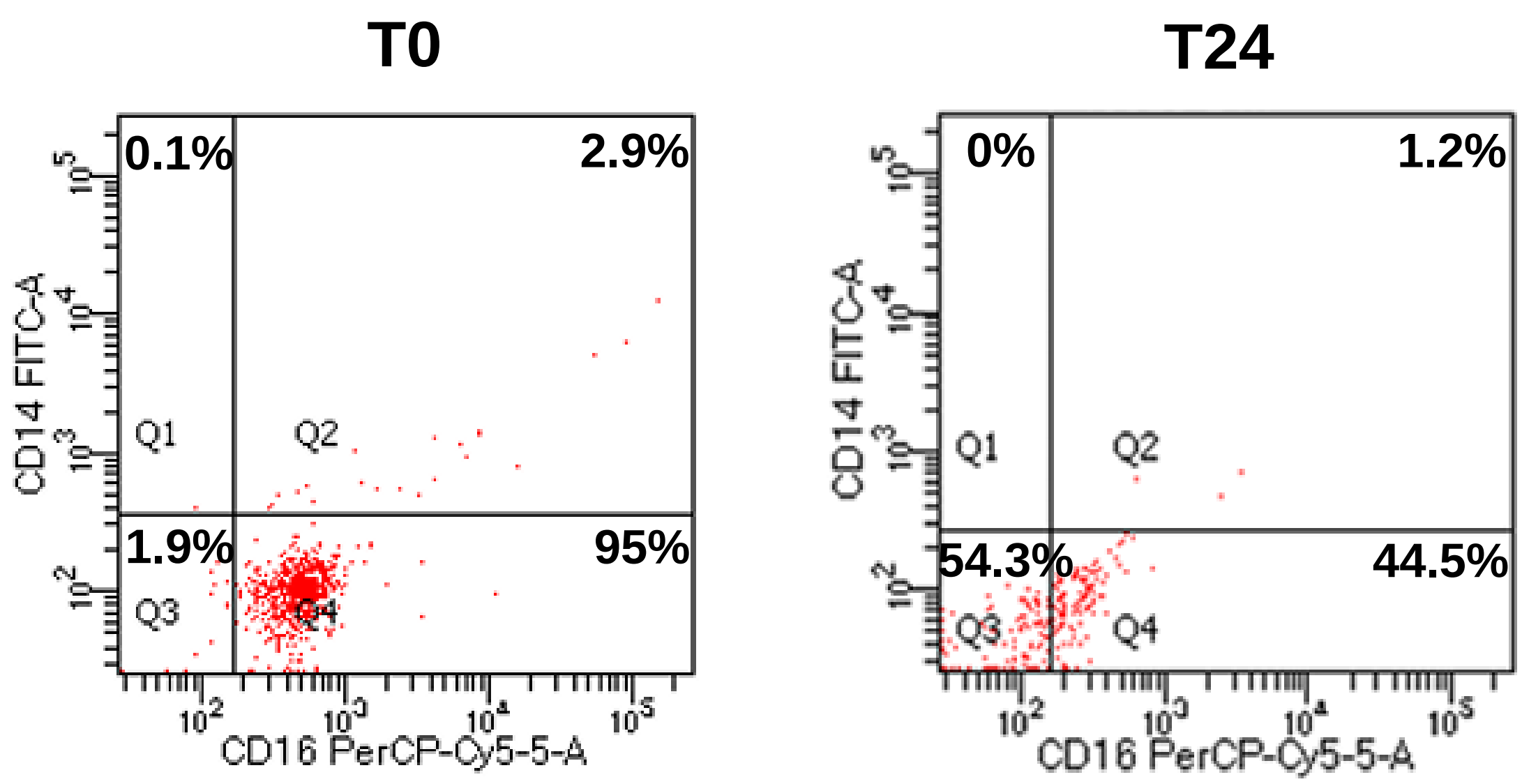

Supplementary Figure S1. Flow cytometry analysis of human neutrophils purity.

Human neutrophil preparations were analyzed immediately after their isolation (T0) and after 24 h of culture by flow cytometry using CD14 and CD16 surface markers. Representative dot plots showing CD14 and CD16 expression after exclusion of debris. Neutrophils were identified as $CD16^{+}CD14^{-}$ cells, whereas $CD14^{+}$ cells were considered contaminating monocytes. The percentage of $CD14^{+}$ cells was 3.0% immediately after purification and 1.2% after 24 h of culture, confirming the high purity of the neutrophil preparations used throughout the study.

Supplementary Material

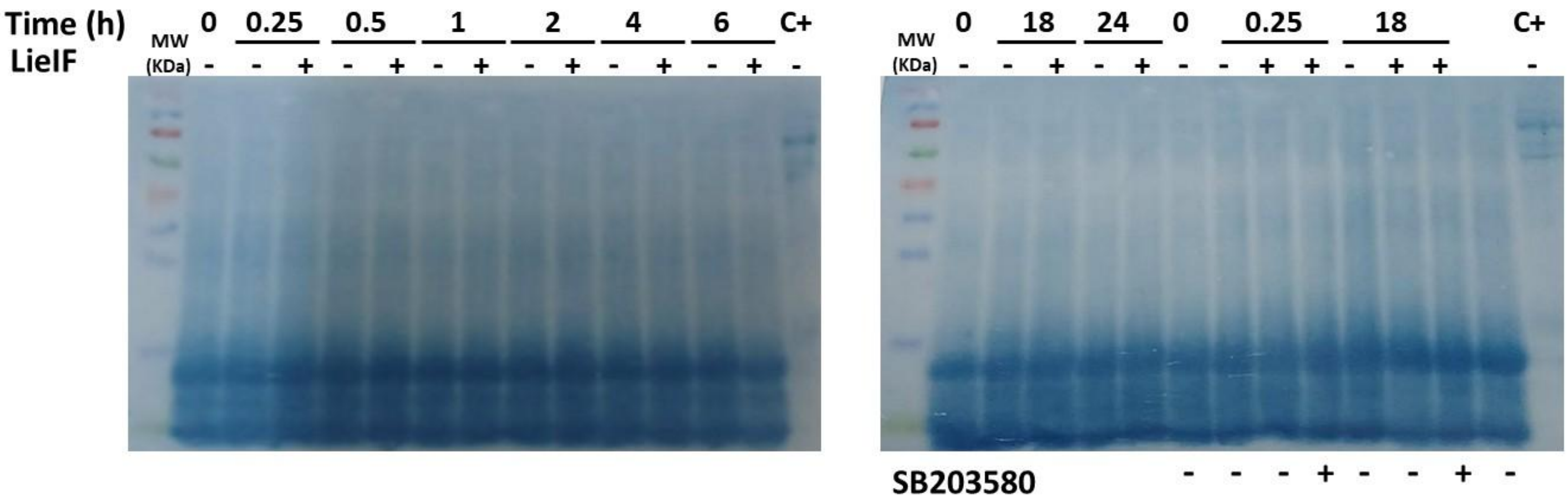

Supplementary Figure S2. Representative Amido Black staining as control of equal total protein loading. Representative Amido Black staining of PVDF membranes investigating MAPK (p38, ERK1/2 and JNK) and PI3K/activations. Similar total protein staining profiles were observed across conditions, supporting comparable protein loading and transfer efficiency.

Human neutrophils were incubated in the absence or presence of recombinant LieIF protein (2.5 µg/mL) for the indicated time points. Total cellular proteins (30 µg per lane) were resolved by SDS–PAGE and analyzed by Western blotting. Time-course analysis of kinases phosphorylations versus their total forms in unstimulated and LieIF-stimulated neutrophils at the indicated time points.

Neutrophils were pretreated with the pharmacological inhibitor of p38 MAPK (SB203580) for 1h prior to LieIF stimulation. Phosphorylation of p38 MAPK was analyzed at early (0.25 h) and late (18 h) time points. LPS-stimulated RAW 264.7 cells were used as a positive control for MAP kinases activation.

Supplementary Material

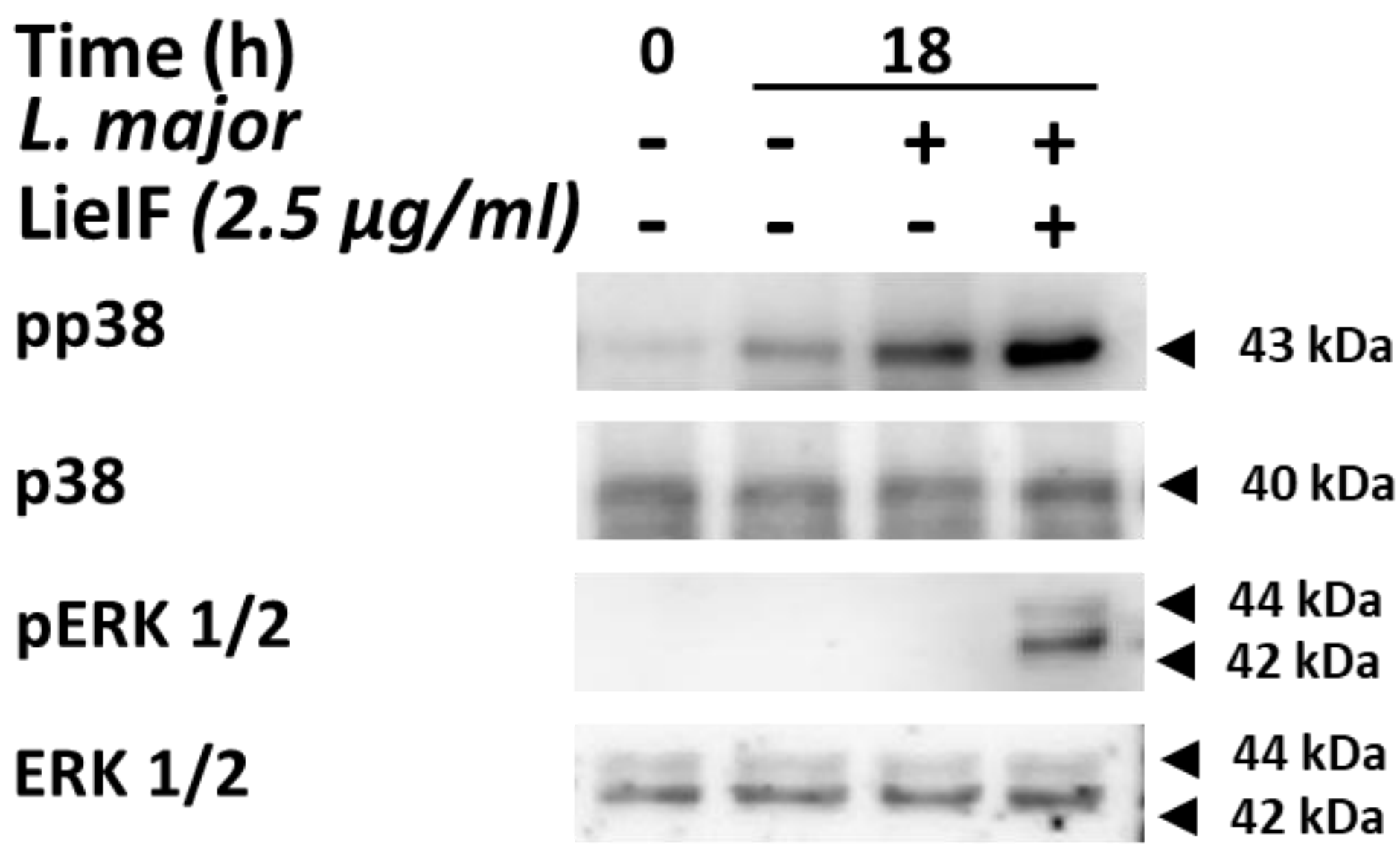

Supplementary Figure S3. LieIF-induced p38 MAPK activation is maintained in *Leishmania major*-infected human neutrophils.

Human neutrophils were used under different experimental conditions: untreated neutrophils cultured for 18 h (PMN), *Leishmania major* (MHOM/TN/2012/Empa-12)-infected neutrophils, and LieIF-pretreated infected neutrophils. Infection was performed using a parasite-to-neutrophil ratio of 10:1, as previously described (Oualha et al., 2019). For LieIF-pretreated conditions, neutrophils were incubated with recombinant LieIF prior to infection and subsequently maintained under infection conditions for a total experimental period not exceeding 18 h. Phosphorylation of p38 MAPK and ERK1/2 was analyzed by Western blotting. Total p38 MAPK and total ERK1/2 were used for normalization of phosphorylated protein levels. The results show that *L. major* infection induced p38 MAPK phosphorylation, which was further enhanced in LieIF-pretreated infected neutrophils, whereas no detectable ERK1/2 phosphorylation was observed under these conditions.
